# Circulating Immune-Related Biomarkers in Egyptian Women with Early Breast Cancer: An Exploratory Case–Control Study Across IHC-Defined Clinicopathological Subgroups

**DOI:** 10.3390/cells15201849

**Published:** 2026-10-12

**Authors:** Rania Zaki, Auhood Nassar, Rania A. Al Najar, Hussein Sabit, Borros Arneth, Asmaa A. El Leithy

**Affiliations:** 1 Department of Medical Biotechnology, College of Biotechnology, Misr University for Science and Technology, Giza 12568, Egypt; 2 Virology and Immunology Unit, Cancer Biology Department, National Cancer Institute, Cairo University, Cairo 11796, Egypt; 3 Breast Cancer Comprehensive Center, Department of Clinical Pharmacy, National Cancer Institute, Cairo University, Cairo 11796, Egypt; 4 Molecular Diagnostics, Institute of Laboratory Medicine and Pathobiochemistry, Hospital of the Universities of Giessen and Marburg (UKGM), Philipps University Marburg, Baldingerstr. 1, 35043 Marburg, Germany; 5 Molecular Diagnostics, Institute of Laboratory Medicine and Pathobiochemistry, Hospital of the Universities of Giessen and Marburg (UKGM), Justus Liebig University Giessen, Feulgenstr 12, 35392 Giessen, Germany; 6 College of Biotechnology, Misr University for Science and Technology, Giza 12568, Egypt

**Keywords:** breast cancer, immune checkpoints, ICOSLG, LAG-3, TIM-3, TNFRSF-14 (HVEM), CX3CL1, fractalkine, M-CSF, FGF-21, immuno-oncology

## Abstract

Background: Breast cancer (BC) progression depends on immune dysregulation and complex interactions between tumor cells and host immune components. Soluble immune checkpoints, growth factors, chemokines, and cytokines are promising circulating biomarkers; however, their clinical relevance across IHC-defined clinicopathological subgroups in Egyptian BC patients remains poorly characterized. Methods: Seventy Egyptian women with pathologically confirmed early-stage BC and 20 healthy controls were recruited for this case–control study. Patients were classified into IHC-defined clinicopathological groups. Eleven circulating proteins (ICOSLG, LAG-3, TIM-3, TNFRSF-14 (HVEM), FGF-21, M-CSF, CX3CR1, CCL5, CX3CL1/fractalkine, IFN-A2A, and TGF-β1) were quantified by sandwich ELISA. Associations with IHC-defined clinicopathological subgroups was assessed using univariate and multivariate analyses. Biomarker performance was assessed by receiver operating characteristic (ROC) analysis and heatmaps. Results: The cohort comprised Luminal A-like (*n* = 20), Luminal-HER2-like (*n* = 18), Luminal B-like (*n* = 16), HER2-positive (*n* = 9), and TNBC (*n* = 7) groups. BC cases had significantly higher circulating levels of ICOSLG, TIM-3, FGF-21, M-CSF, CX3CR1, CCL5, CX3CL1/fractalkine, TGF-β1, and TNFRSF-14 (HVEM), and significantly lower levels of LAG-3 than healthy controls (all *p* < 0.05). Statistically significant subtype-related differences were observed for ICOSLG, LAG-3, FGF-21, M-CSF, CX3CL1/fractalkine, TGF-β1, and TNFRSF-14 (HVEM). In multivariate logistic regression, Spearman rank correlation analysis confirmed that CX3CL1/fractalkine and TNFRSF-14 (HVEM) were independently associated with IHC-defined clinicopathological subgroups and can distinguish Luminal-HER2 BC from the Luminal A subtype [CX3CL1/Fractalkine: AUC of 0.789 (95% CI: 0.643–0.935), *p* = 0.002; TNFRSF-14 (HVEM): AUC of 0.858 (95% CI: 0.735–0.981), *p* < 0.001). For HER2 positivity, a combined LAG-3/CCL5 ROC for HER2-positive vs. HER2-negative was yielded AUC = 0.775 (95% CI: 0.659–0.882). Conclusions: Early BC appears to trigger significant systemic immune dysregulation, marked by altered circulating levels of immune checkpoints, inflammatory mediators, and growth factors. CX3CL1/fractalkine and TNFRSF-14 (HVEM) can independently discriminate Luminal-HER2 BC from the Luminal A subtype and may serve as potential biomarkers for disease stratification and precision immuno-oncology.

## 1. Introduction

BC is the most prevalent malignancy among women worldwide. Despite advances in screening, molecular classification, and targeted therapies, BC remains a leading cause of cancer-related morbidity and mortality [1]. BC pathogenesis is shaped by intrinsic genetic alterations and interactions between tumor cells and host immune cells. The tumor-immune microenvironment plays a vital role in triple-negative BC (TNBC) by influencing immune responses and potentially affecting disease progression [2]. Membrane-bound immune checkpoints, including programmed death-1 (PD-1), programmed death ligand-1 (PD-L1), cytotoxic T-lymphocyte-associated protein-4 (CTLA-4), and lymphocyte activation gene-3 (LAG-3), have been extensively investigated in cancer immunology. Recently, soluble forms of these receptors have been identified as relevant to cancer-associated immune dysregulation. These soluble receptors may reflect systemic immune status beyond the local tumor microenvironment (TME) and have potential as noninvasive diagnostic, prognostic, and treatment monitoring biomarkers [3,4].

Abnormal expression and activity of the chemokine CCL5 and its receptor CCR5 have been observed in both blood cancers and solid tumors, suggesting a significant role for the CCL5/CCR5 axis in cancer progression [5]. In parallel, epithelial–mesenchymal transition (EMT) not only initiates tumor metastasis but also confers additional clinically relevant traits on cancer cells [6]. Collectively, these mediators form an interconnected signaling network linking systemic immunity to tumor biology.

Recent studies show that patients with early-stage BC exhibit systemic immune alterations, including elevated levels of circulating immune checkpoint molecules, chemokines, and growth factors. However, biomarker profiles vary across BC molecular subtypes, with levels differing by subtype [7]. Direct data on how soluble immune checkpoints relate to specific molecular subtypes and other clinicopathological features are just beginning to emerge [8]. These knowledge gaps continue to hinder the development of precise, subtype-specific immune profiles and the identification of biomarkers that advance precision oncology and more accurate disease stratification. The immune microenvironment and clinical trajectory of BC vary across molecular subtypes, including TNBC, Luminal-HER2 BC, HER2-positive, Luminal A, and Luminal B. Immunological subtyping may provide insights into tumor immunobiology and facilitate the identification of biomarkers with diagnostic and therapeutic significance, particularly across these molecular subtypes [9].

BC immunobiology varies across ethnic and geographic populations due to genetic predisposition, hormonal milieu, environmental exposures, and tumor molecular characteristics [10]. Immunological markers in Egyptian BC patients are beginning to emerge, but comprehensive data on circulating immune checkpoint molecules and their interactions with inflammatory and growth-related mediators remain limited [11]. Investigating immune biomarkers in Egyptian BC patients provides insight into how immune molecules, especially cytokines, are affected by treatments such as resiquimod [12]. The soluble immune checkpoint molecules assessed were inducible T cell co-stimulator ligand (ICOSLG), lymphocyte activation gene-3 (LAG-3), T-cell immunoglobulin and mucin domain-3 (TIM-3), and tumor necrosis factor receptor superfamily member 14 (TNFRSF14), also known as herpes virus entry mediator TNFRSF-14 (HVEM). The role of CXCL1 in the interaction between endocrine-resistant BC and fibroblasts was examined, with a focus on disease-relevant chemokines [13]. The prognostic role of interferon-γ (IFN-γ), a proinflammatory cytokine, was significant mainly in basal-like BC, suggesting that its relevance varies by molecular subtype [14].

Population-based data show that the mean age of BC diagnosis in Egypt is approximately 50 years, with a significantly later stage at presentation (<5% at Stage I vs. ~45% at Stage III) compared to Western populations [15]. This younger, pre-menopausal demographic is prone to more aggressive tumor biology. Because circulating immune markers are understudied in the Egyptian population [11,12], characterization of a comprehensive biomarker panel is essential to establish accurate, population-specific data.

To date, no study has simultaneously evaluated this 11-protein circulating panel in Egyptian patients with early-stage BC while integrating IHC-defined clinicopathological subgrouping, multivariable biomarker analyses, and public multi-omics context. This study addresses this gap and aims to characterize the immunological heterogeneity of BC and assess the potential of these biomarkers to advance precision immuno-oncology.

## 2. Study Design and Participants

This case–control study investigated circulating immune biomarkers in Egyptian women with early BC. A total of 70 patients with histopathologically confirmed BC and 20 age-matched healthy female controls were recruited. Patients with BC were recruited consecutively from the BC Hospital at the NCI, Cairo University, Egypt, from January 2026 to May 2026. Healthy controls were women who were apparently healthy and had no history of malignancy, autoimmune disease, chronic inflammatory disorders, or active infection. Clinical and pathological characteristics, including age, menopausal status, tumor size, histological grade, hormone receptor status (ER and PR), HER2 status, and family history of BC, were obtained from medical records.

## 3. Ethics Committee Approval

The study protocol was reviewed and approved by the Institutional Review Board (IRB) under approval number CB2509-102-120-202. All participants provided informed written consent in accordance with the Declaration of Helsinki and its amendments.

## 4. Methods

### 4.1. Sample Collection

Whole-blood samples were collected aseptically from all patients with BC and healthy controls before any systemic chemotherapy was initiated and before surgery. Plasma was immediately separated and stored at −80 °C until analysis.

### 4.2. Biomarker Measurements

Eleven circulating immune biomarkers were measured in plasma from 70 early BC patients and compared with those from 20 healthy controls using commercially available sandwich enzyme-linked immunosorbent assay (ELISA) kits according to the manufacturers’ instructions (BT Laboratory, Shanghai, China). To ensure high reproducibility and quality control, the manufacturer’s provided standards and blank to construct the standard curve and monitor inter-assay variability. We selected this panel to get a clear picture of the systemic tumor-immune biology. The biomarkers include; soluble checkpoints (ICOSLG, LAG-3, TIM-3, TNFRSF-14 (HVEM)), growth factors (FGF-21, M-CSF), chemokine trafficking (CX3CR1, CCL5, CX3CL1), and immunoregulatory cytokines (IFN-α2a, TGF-β1). The panel design is based on established evidence from prior literature showing checkpoint dysregulation in early BC, altered macrophage pathways, and how chemokines and TGF-β drive immune regulation [3,7,16,17].

### 4.3. Determination of Soluble Immune Checkpoint Levels

Using the commercially available ELISA kits (BT Laboratory, Shanghai, China), the levels of soluble immune checkpoint molecules, including ICOSLG (Cat. No. E5446Hu), LAG-3 (Cat. No. E5606Hu), TIM-3 (Cat. No. E7264Hu), and TNFRSF-14 (HVEM) (Cat. No. E6641Hu), were measured using quantitative sandwich ELISA kits according to the manufacturer’s instructions.

### 4.4. Determination of Growth Factors, Chemokines, and Cytokine Levels

Levels of growth factors, including FGF-21 (Cat. No. E1983Hu) and M-CSF (Cat. No. E0127Hu); chemokines, including CX3CR1 (Cat. No. E0296Hu), CCL5 (Cat. No. E5009Hu), and CX3CL1/Fractalkine (FKN; Cat. No. E0109Hu); and cytokines, including IFN-α2a (Cat. No. E4235Hu) and TGF-β1 (Cat. No. E0134Hu), were measured. Absorbance was measured at 450 nm using a microplate reader, and biomarker concentrations were quantified using an assay-specific standard curve per the manufacturer’s protocol.

### 4.5. Pathological Assessment and IHC-Defined Clinicopathological Subgrouping

Pathological status was determined by routine immunohistochemistry (IHC) following standard clinical guidelines. Estrogen receptor (ER) and progesterone receptor (PR) positivity were defined by ≥1% positive tumor cell nuclear staining. HER2 positivity was defined as an IHC score of 3+. Tumors were categorized into; Luminal A: ER-positive and/or PR-positive, HER2-negative, and low proliferation (Ki-67 < 14% or low tumor grade), Luminal B: ER-positive and/or PR-positive, HER2-negative, and high proliferation (Ki-67 ≥ 14% or high tumor grade), HER2 Enriched: ER-negative, PR-negative, and HER2-positive, Triple Positive Breast Cancer (Luminal-HER2 BC): ER-positive and/or PR-positive, and HER2-positive), and Triple-Negative Breast Cancer (TNBC): negative for ER, PR, and HER2.

### 4.6. Study Outcomes

This study compared the circulating plasma concentrations of the 11 measured proteins between early BC cases and healthy controls, as well as across IHC-defined clinicopathological groups.

### 4.7. Statistical Analysis

IBM SPSS Statistics (version 26, IBM Corp., Armonk, NY, USA) was used for data management and statistical analyses. Normality of numerical variables was assessed, and results were reported as mean ± standard deviation or median (range), as appropriate. Categorical variables were reported as frequencies and percentages. Comparisons between two independent groups were made using the Mann–Whitney U test, and comparisons among multiple groups were made using the Kruskal–Wallis test, with Bonferroni-adjusted post hoc pairwise comparisons as needed.

Spearman’s rank correlation coefficient (ρ) was used to assess correlations between biomarkers and clinicopathologic parameters. Multiple logistic regression analysis was used to determine independent predictors. The forward LR variable selection method was used, and all variables that were statistically significant in univariate analyses were included in the model. Odds ratios (ORs) were calculated for significant variables in the final step of the logistic regression. To assess discrimination performance, ROC analysis was performed. Accuracy was measured by the area under the ROC curve (AUC). An area close to 1.0 indicates a perfect test; an area close to 0.5 indicates a worthless test. To visualize global expression patterns of these biomarkers and identify coordinated immune signatures associated with BC, a heatmap visualization and systems immunology analyses were performed in R version 4.5.1. All tests were conducted two-sided; *p*-values < 0.05 were deemed statistically significant, and a 95% confidence level was used.

### 4.8. Multivariable Circulating Immune-Profile Analysis

Multivariable analysis was performed using the complete 11-biomarker dataset from the 70 patients with BC to investigate the immune variation beyond single-biomarker comparison. Transformation performance was evaluated before multivariable analysis because of the right-skewed distribution of the circulating biomarkers. Yeo–Johnson transformation produced the greatest overall reduction in skewness and was then applied to the biomarker concentrations. The transformed values were then standardized to z-scores (mean = 0, standard deviation = 1). Principal component analysis (PCA) was performed on the standardized 11-biomarker dataset. The proportion of variance was calculated, and biomarker loadings were examined to characterize the biological composition of the leading components. PCA scores were visualized according to the conventional BC clinicopathological subtypes.

Associations between the leading principal-component scores and clinicopathological characteristics were assessed using non-parametric tests. Wilcoxon rank-sum tests were used for binary variables, including ER, PR, HER2, histological grade, and lymph-node status, whereas Kruskal–Wallis tests were used for menopausal status and BC subtype. Multiple testing across clinicopathological variables was controlled using the Benjamini–Hochberg false-discovery rate (FDR). Dunn’s test with Benjamini–Hochberg adjustment was used for post-hoc comparisons among molecular subtypes when the global Kruskal–Wallis test was significant. Effect sizes were estimated using Wilcoxon effect-size r for two-group comparisons and Kruskal–Wallis eta-squared (η^2^[H]) for subtype comparisons.

## 5. Results

The current study included 70 newly diagnosed women with early BC and 20 age-matched control participants. The mean age among patients was 50 ± 12 years. Regarding menopausal status, 50.0% of patients were premenopausal, 45.7% were postmenopausal, and 4.3% were perimenopausal. The most common tumor grade was grade II (62.9%), followed by grade III (37.1%). Tumor size was as follows: T1 (17.1%), T2 (58.6%), and T3 (24.3%). Lymph node involvement was present in 78.6% of patients. ER positivity was observed in 75.7% of participants, and PR positivity in 71.4%. Human epidermal growth factor receptor 2 (HER2) positivity/amplification was identified in 41.4% of patients. Based on the IHC-defined clinicopathological subgrouping, patients were grouped as Luminal A (28.6%), Luminal-HER2 BC (25.7%), Luminal B (22.9%), HER2-positive (12.9%), and TNBC (10.0%). Patient characteristics are outlined in Table 1.

### 5.1. Comparison of Circulating Biomarkers Between Early BC Patients and Healthy Controls

Significantly altered levels of circulating immune checkpoint molecules, growth factors, chemokines, cytokines, and dual immune regulatory proteins were found in patients with early BC compared with healthy controls, as shown in Table 2.

### 5.2. Soluble Immune Checkpoint Molecules

Patients with early BC showed significantly increased plasma levels of ICOSLG and TIM-3, and significantly decreased plasma levels of LAG-3 compared with healthy controls (all *p* < 0.001). Specifically, the median plasma levels of ICOSLG increased from 510.25 pg/mL in controls to 697.48 pg/mL in patients with BC, whereas the median level of TIM-3 increased from 217.68 pg/mL to 350.40 pg/mL. LAG-3 significantly decreased in patients from 6.37 pg/mL in healthy individuals to 3.24 pg/mL. In the case of TNFRSF-14 (HVEM), significantly elevated plasma levels in patients with early BC were observed compared to healthy controls (266.112 vs. 219.73 pg/mL, *p* = 0.002).

### 5.3. Growth Factor

Growth factor levels of both measured molecules were significantly higher than those in healthy controls. In particular, median plasma levels of FGF-21 increased from 57.49 pg/mL in healthy controls to 68.36 pg/mL in patients (*p* < 0.001), while median M-CSF levels increased from 957.87 pg/mL to 1635.51 pg/mL (*p* < 0.001).

### 5.4. Chemokines

Circulating levels of all measured chemokines were significantly higher in patients with early BC than in healthy controls. Specifically, median plasma levels of CX3CR1 (435.15 vs. 277.44 pg/mL), CCL5 (484.26 vs. 284.70 pg/mL), and CX3CL1/fractalkine (3.47 vs. 2.96 pg/mL) were significantly higher in patients than in healthy subjects (all *p* < 0.001).

### 5.5. Cytokines

Among the cytokines analyzed, median TGF-β1 levels were significantly higher in BC patients than in healthy controls (451.78 vs. 404.60 pg/mL; *p* = 0.031). In contrast, no statistically significant difference was observed in plasma IFN-α2a levels (*p* = 0.570).

**Figure 1:**
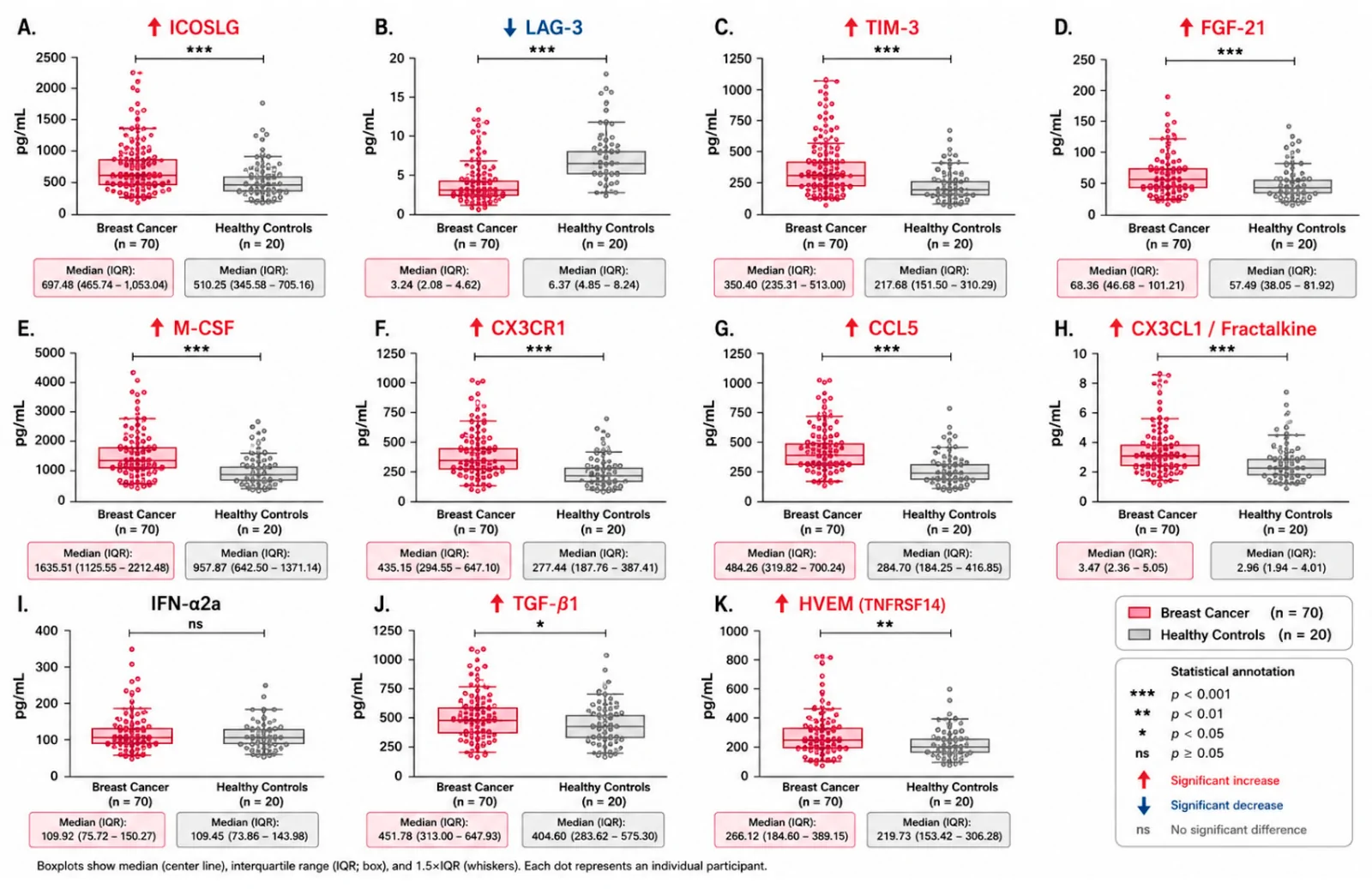
Comparison of circulating immune biomarkers between early-stage BC patients and healthy controls. (**A**) ICOSLG; (**B**) LAG-3; (**C**) TIM-3; (**D**) FGF-21; (**E**) M-CSF; (**F**) CX3CR1; (**G**) CCL5; (**H**) CX3CL1 (fractalkine); (**I**) IFN-α2a; (**J**) TGF-β1; and (**K**) HVEM (TNFRSF14).

**Figure 2:**
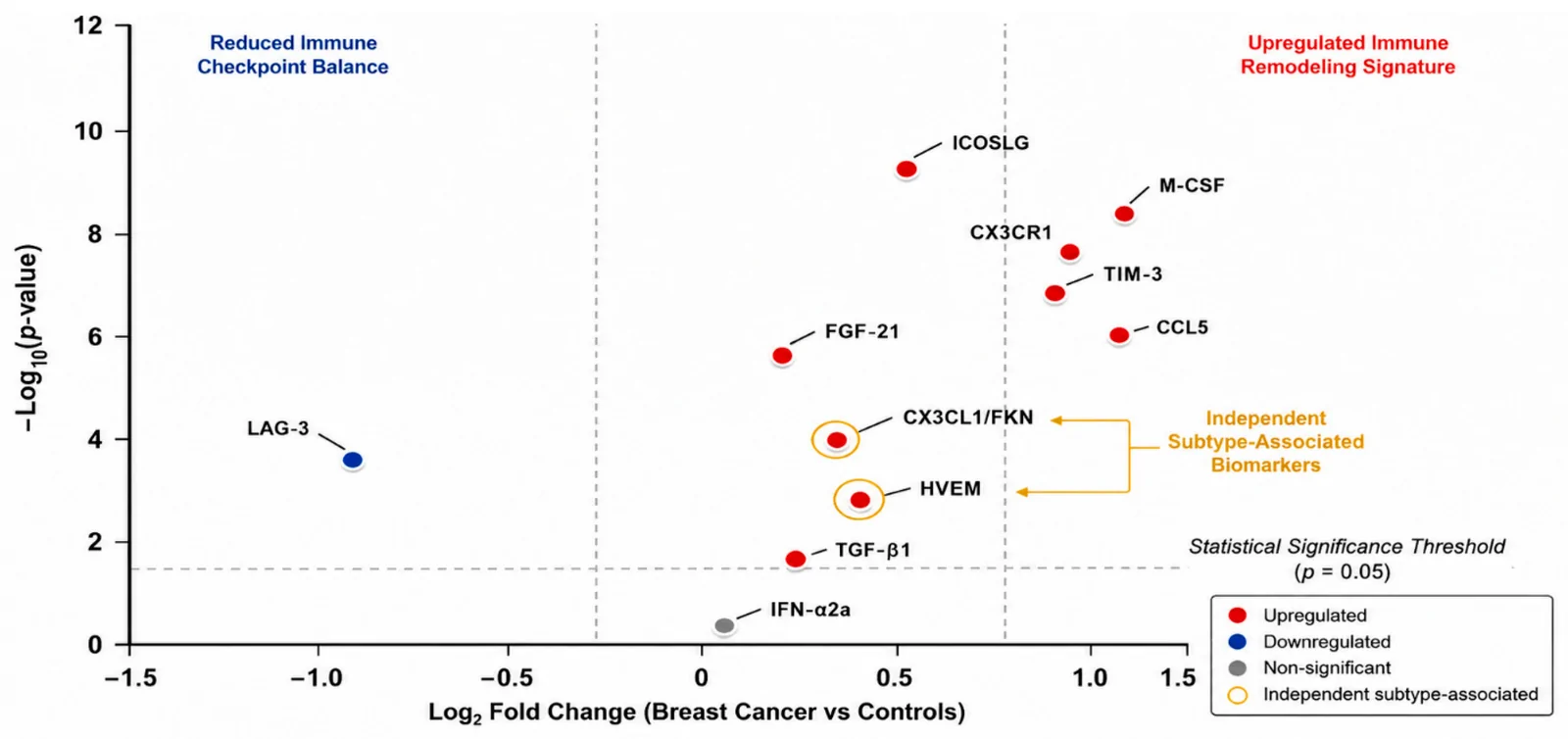
A volcano plot was created using log2 fold change and statistical significance values to provide an integrated overview of biomarker alterations.

The box plots of circulating immune biomarkers in Egyptian patients with early BC compared with healthy controls are shown in Figure 1. Volcano plot analysis also demonstrated widespread immune dysregulation in BC, as shown in Figure 2. Elevated plasma concentrations of ICOSLG, TIM-3, FGF-21, M-CSF, CX3CR1, CCL5, CX3CL1/Fractalkine, TGF-β1, and TNFRSF-14 (HVEM) was detected, whereas LAG-3 was significantly lower. The levels of IFN-α2a did not differ significantly between the two groups. Supplementary Figure S1 displays the distributions of all 11 circulating proteins across healthy controls and IHC-defined clinicopathological subgroups (healthy controls *n* = 20; Luminal A-like *n* = 20; Luminal B-like *n* = 16; Luminal-HER2-like *n* = 18; HER2-positive/non-luminal *n* = 9; TNBC *n* = 7).

### 5.6. Association of Circulating Immune Biomarkers with BC IHC-Defined Clinicopathological Subgroups

**Figure 3:**
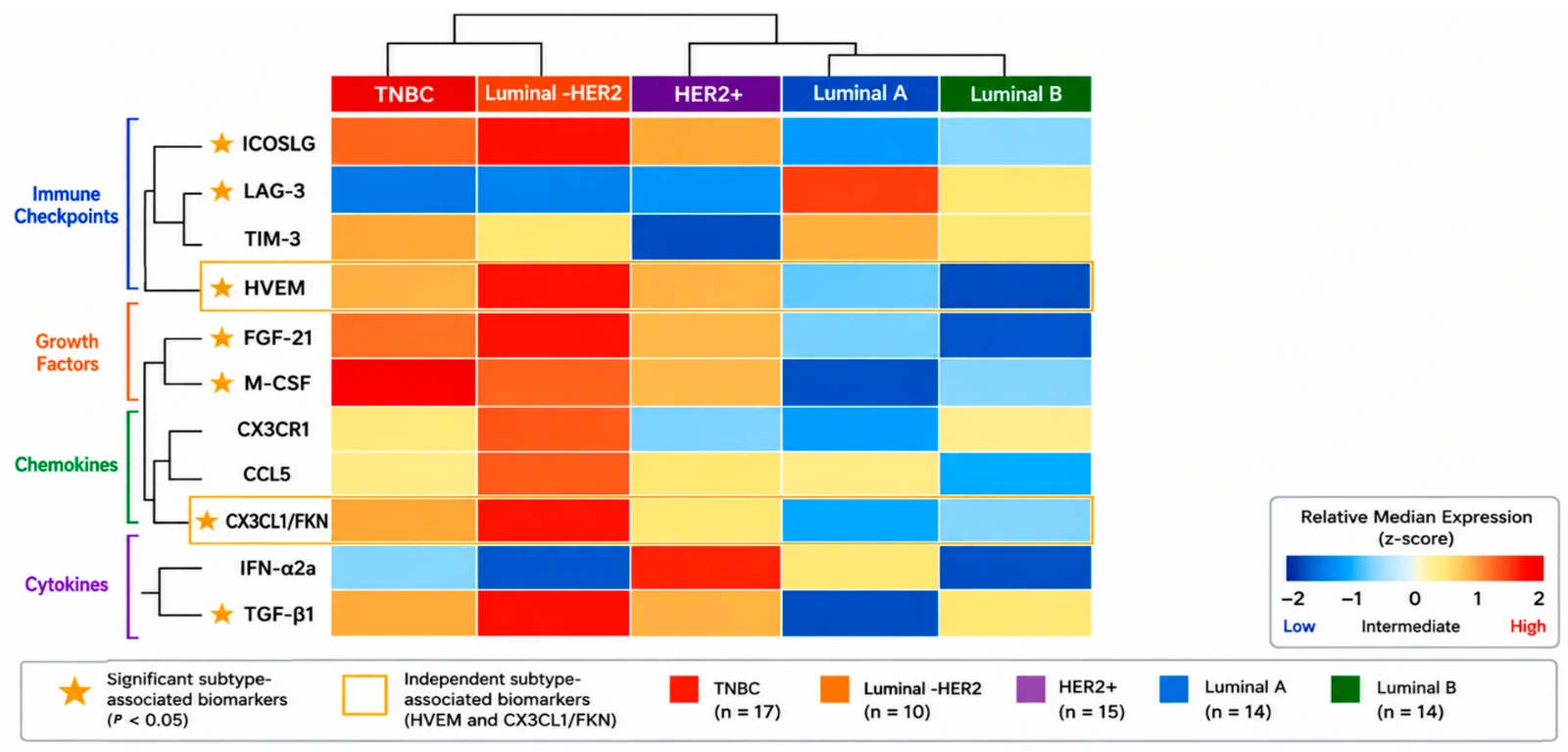
Median circulating levels of immune biomarkers in BC patients, stratified by molecular subtype. Colored circles indicate Z-score-normalized medians for each row.

Significant differences in circulating levels of immune biomarkers were found in BC IHC-defined clinicopathological subgroups. Statistically significant subtype-specific changes were detected in ICOSLG (*p* = 0.007), LAG-3 (*p* = 0.012), FGF-21 (*p* < 0.001), M-CSF (*p* = 0.003), CX3CL1/Fractalkine (*p* = 0.016), TGF-β1 (*p* < 0.001), and TNFRSF-14 (HVEM) (*p* = 0.001). Notably, no statistically significant differences were found in the levels of circulating TIM-3, CX3CR1, CCL5, and IFN-α2a across IHC-defined clinicopathological subgroups. Table 3 summarizes the circulating levels of the immune biomarkers across BC IHC-defined clinicopathological subgroups. A heatmap illustrating the median circulating levels of immune biomarkers in BC patients stratified by IHC-defined clinicopathological subgroups is presented in Figure 3.

### 5.7. Post Hoc Pairwise Analysis of IHC-Defined Clinicopathological Subgroups

Post hoc pairwise comparisons were carried out for immune biomarkers that showed significant differences across subtypes using the Kruskal–Wallis test. However, after Bonferroni correction, a few of these differences remained significant, as presented in Table 4. Of note, patients with Luminal-HER2 BC had significantly elevated levels of FGF-21, CX3CL1/Fractalkine, and TNFRSF-14 (HVEM) relative to those with Luminal A BC (adjusted *p*-values < 0.05 for all). However, the differences between Luminal A and Luminal-HER2 BC for ICOSLG, LAG-3, M-CSF, and TGF-β1 became nonsignificant after correction. Overall, these results highlight FGF-21, CX3CL1/Fractalkine, and TNFRSF-14 (HVEM) as key biomarkers distinguishing Luminal-HER2 BC from Luminal A BC.

### 5.8. Multivariate Logistic Regression Analysis

**Figure 4:**
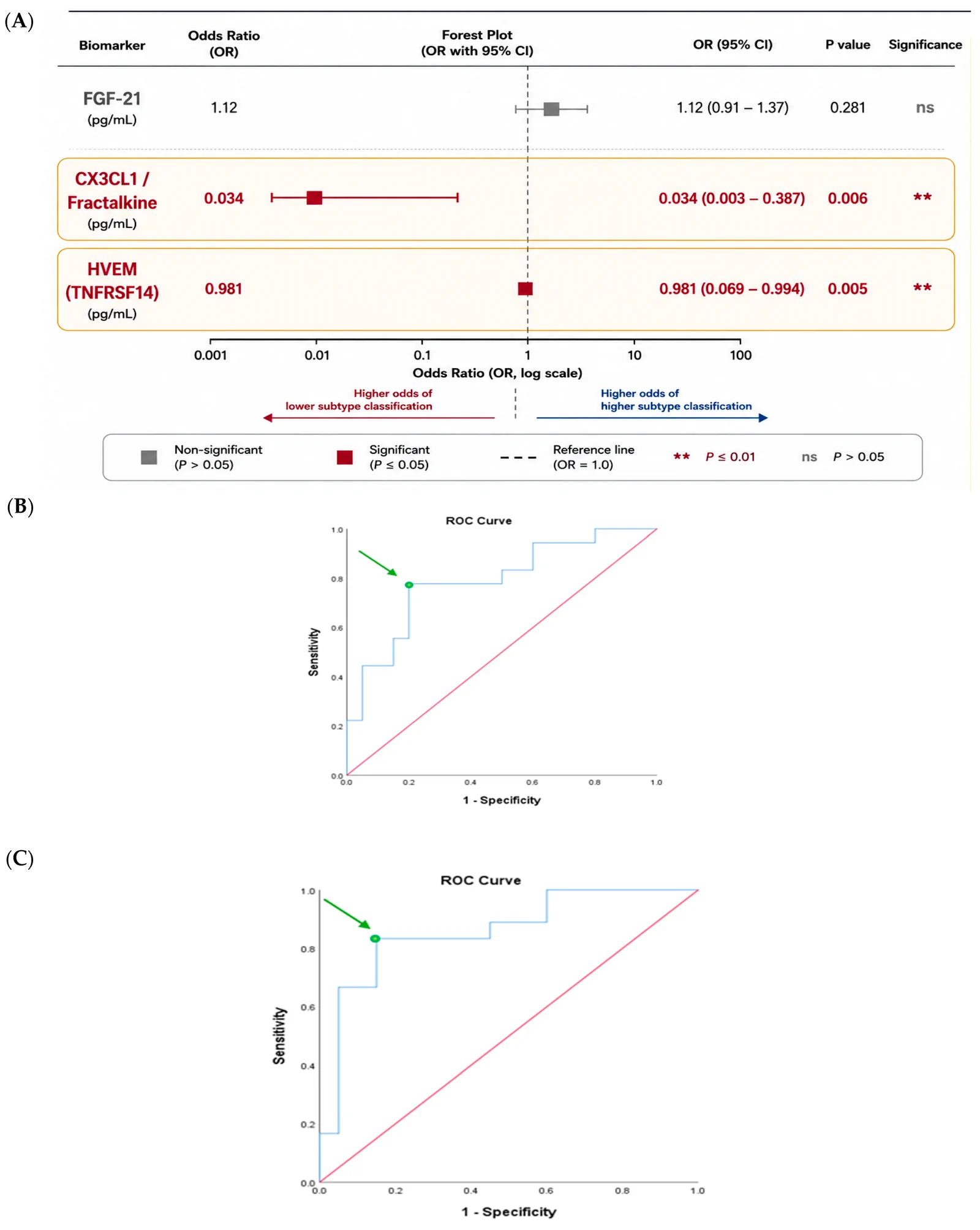

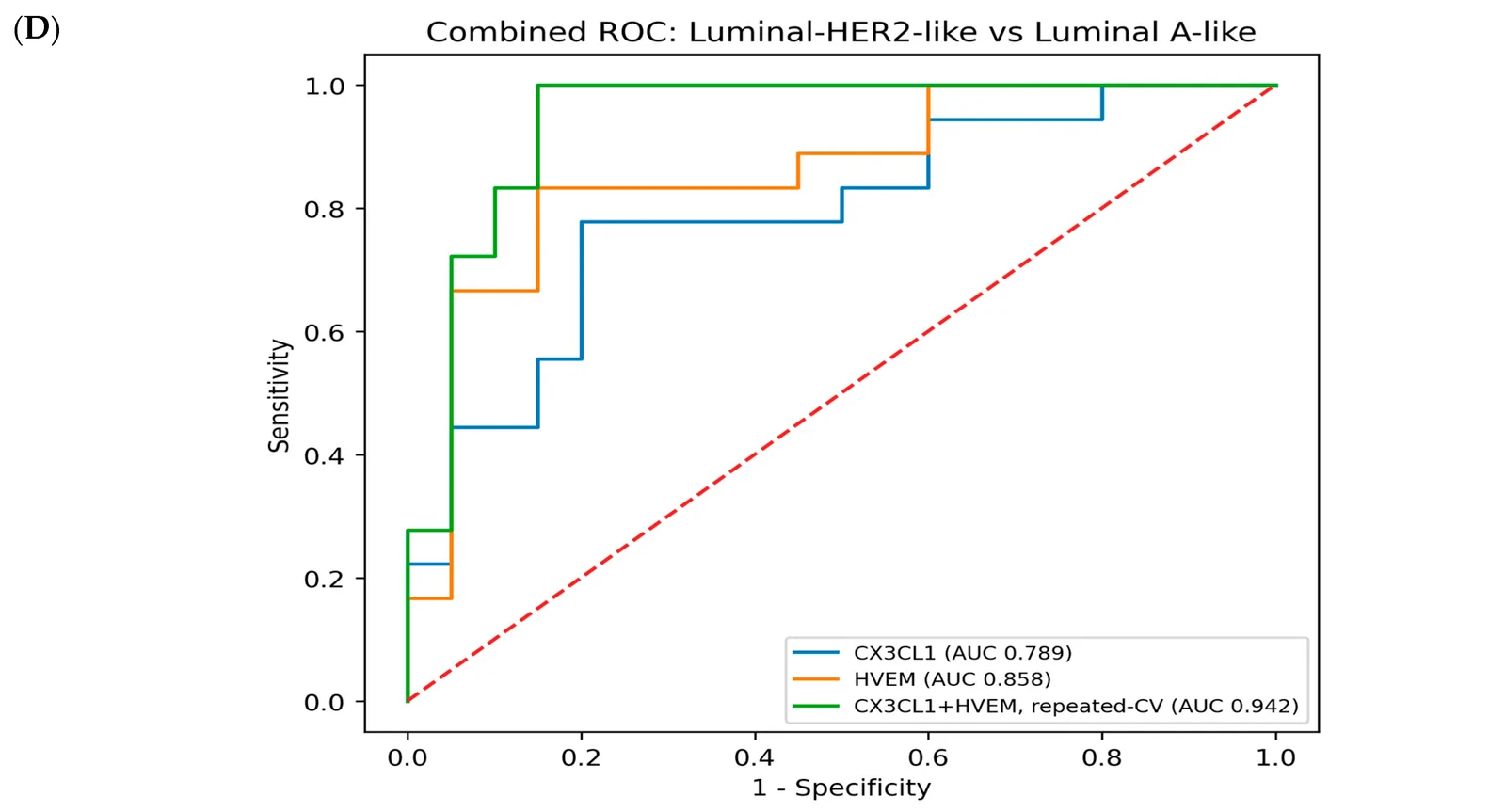
(**A**) Forest plot illustrating the multivariate logistic regression analysis of circulating candidate proteins evaluated in the Luminal-HER2 BC versus Luminal A BC. (**B**,**C**) Single-marker ROC curves for CX3CL1-FKN (The green dot represents the optimal cutoff value of 3.65, with a sensitivity of 77.8% and a specificity of 80.0%) and TNFRSF-14 (HVEM) (The green dot indicates the optimal cutoff value of 276.32, with a sensitivity of 83.3% and a specificity of 85.0%). (**D**) Combined-model ROC for CX3CL1 + TNFRSF-14 (HVEM). The combined AUC was 0.942 (95% CI: 0.849–1.000); paired AUC increments versus the individual markers did not reach *p* < 0.05.

Multivariate logistic regression analysis incorporated the significant biomarkers in the comparison between the luminal A and Luminal-HER2 BC groups following adjustment for covariates (FGF-21, CX3CL1-FKN, and TNFRSF-14 (HVEM) to account for potential confounding factors. In the final stage of analysis, only CX3CL1-FKN and TNFRSF-14 (HVEM) demonstrated a significant independent association with the IHC-defined clinicopathological subgrouping, suggesting that these biomarkers serve as primary discriminators. The odds ratio (OR) for CX3CL1/fractalkine was 0.034 (95% confidence interval [CI] 0.003–0.387, *p* = 0.006), whereas the OR for TNFRSF-14 (HVEM) was 0.981 (95% CI 0.969–0.994, *p* = 0.005), as detailed in Table 5 and Figure 4A.

### 5.9. Discriminatory Performance of CX3CL1/Fractalkine and TNFRSF-14 (HVEM) in Distinguishing Luminal-HER2 BC from Luminal A Subtype

Subsequent to a multivariate logistic regression analysis of independent biomarkers for distinguishing Luminal-HER2 BC from the Luminal A subtype, ROC curve analysis demonstrated satisfactory discriminatory performance for both CX3CL1/Fractalkine and TNFRSF-14 (HVEM). The CX3CL1/Fractalkine biomarker exhibited an area under the curve (AUC) of 0.789 (95% confidence interval [CI]: 0.643–0.935, *p* = 0.002), with an optimal cutoff value of 3.65, yielding a sensitivity of 77.8% and a specificity of 80.0%. The TNFRSF-14 (HVEM) biomarker demonstrated superior performance, with an AUC of 0.858 (95% CI: 0.735–0.981, *p* < 0.001) and an optimal cutoff of 276.32, achieving a sensitivity of 83.3% and a specificity of 85.0%, as depicted in Figure 4B,C. Additionally, a combined-model ROC for CX3CL1/TNFRSF-14 (HVEM) predicting Luminal-HER2 vs. Luminal A was evaluated. The combined AUC was 0.942 (95% CI 0.849–1.000); paired AUC increments versus the individual markers did not reach *p* < 0.05, as detailed in Figure 4D.

### 5.10. Correlation Analysis Among Circulating Immune Biomarkers

**Figure 5:**
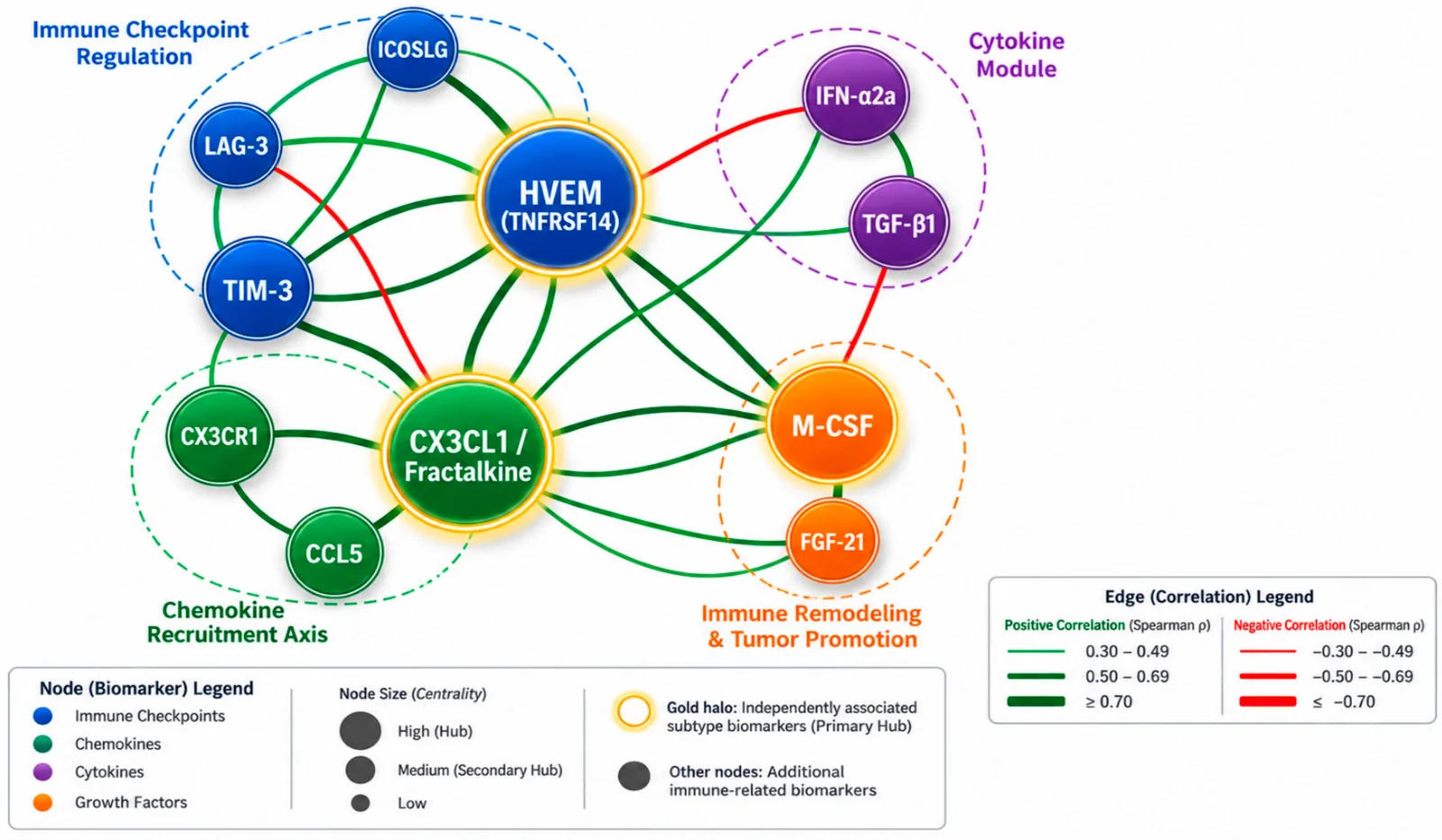
Network of correlations among circulating immune biomarkers in the studied early BC patients.

To better understand the interrelationships among circulating immune mediators, a network analysis based on significant Spearman correlations was conducted. This analysis revealed coordinated interactions among soluble immune checkpoint molecules, growth factors, chemokines, and cytokines in early-stage BC patients. Notably, TNFRSF-14 (HVEM) and CX3CL1/Fractalkine played central roles in this network, corroborating their role in determining heterogeneity of IHC-defined clinicopathological subgroups, as illustrated in Figure 5.

### 5.11. Correlations Between Clinicopathological Features and the Analyzed Biomarkers

The associations between circulating immune biomarkers and clinicopathological features, such as age, menopausal status, tumor grade, tumor size, hormone receptor status, family history, and HER2 expression pattern, are summarized in Table 6. Multivariate logistic regression analysis was conducted, incorporating all significant biomarkers identified at the univariate level (including ICOSLG, LAG-3, FGF-21, M-CSF, CX3CR1-FKN, CCL5, Dual TNFRSF-14 (HVEM), and TGF-β1) to account for covariation. The analysis revealed that only LAG-3 and CCL5 demonstrated a significant independent association with HER2 status.

**Figure 6:**
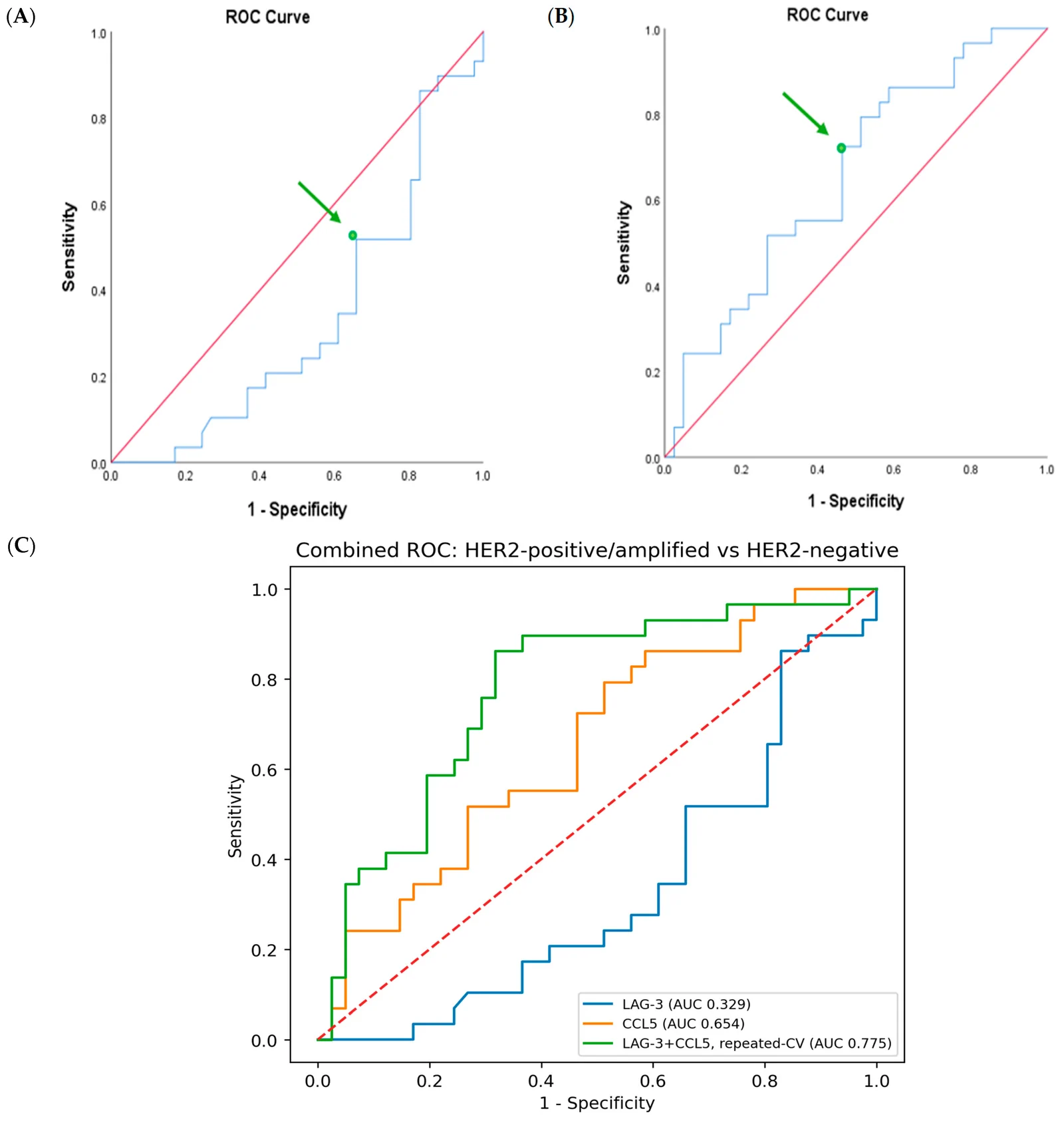
Exploratory ROC curves for distinguishing HER2-positive from HER2-negative BC patients: (**A**) LAG-3, (The green dot indicates the selected cutoff value of 2.96, yielding a sensitivity of 51.7% and a specificity of 34.1%) (**B**) CCL5, (The green dot indicates the selected cutoff value of 464.71, yielding a sensitivity of 72.4% and a specificity of 53.7%) and (**C**) Combined LAG-3 + CCL5 model. The combined AUC was 0.775 (95% CI: 0.659–0.882).

The ROC curve analysis was performed to identify the predictive value of LAG-3 and CCL5 in discriminating HER2-positive from HER2-negative early BC patients. Poor discrimination of LAG-3 was noted, with an AUC of 0.329 (95% CI 0.203–0.456, *p* = 0.016) and (51.7% sensitivity, 34.1% specificity) at a 2.96 pg/mL cut-off point. On the contrary, CCL5 had a better discriminatory power with AUC 0.654 (95% CI 0.526–0.783, *p* = 0.029) and (72.4% sensitivity, 53.7% specificity) at a 464.71 pg/mL cut-off value. Using repeated cross-validation, the combined LAG-3 + CCL5 model yielded an out-of-fold AUC of 0.775 (bootstrap 95% CI 0.659–0.882). This was higher than LAG-3 alone by DeltaAUC = 0.446 (DeLong *p* < 0.001) and higher than CCL5 alone by DeltaAUC = 0.121 (*p* = 0.070) (Figure 6).

### 5.12. Analysis of the Immune Biomarker Landscape Between Healthy Controls and Early BC Patients

**Figure 7:**
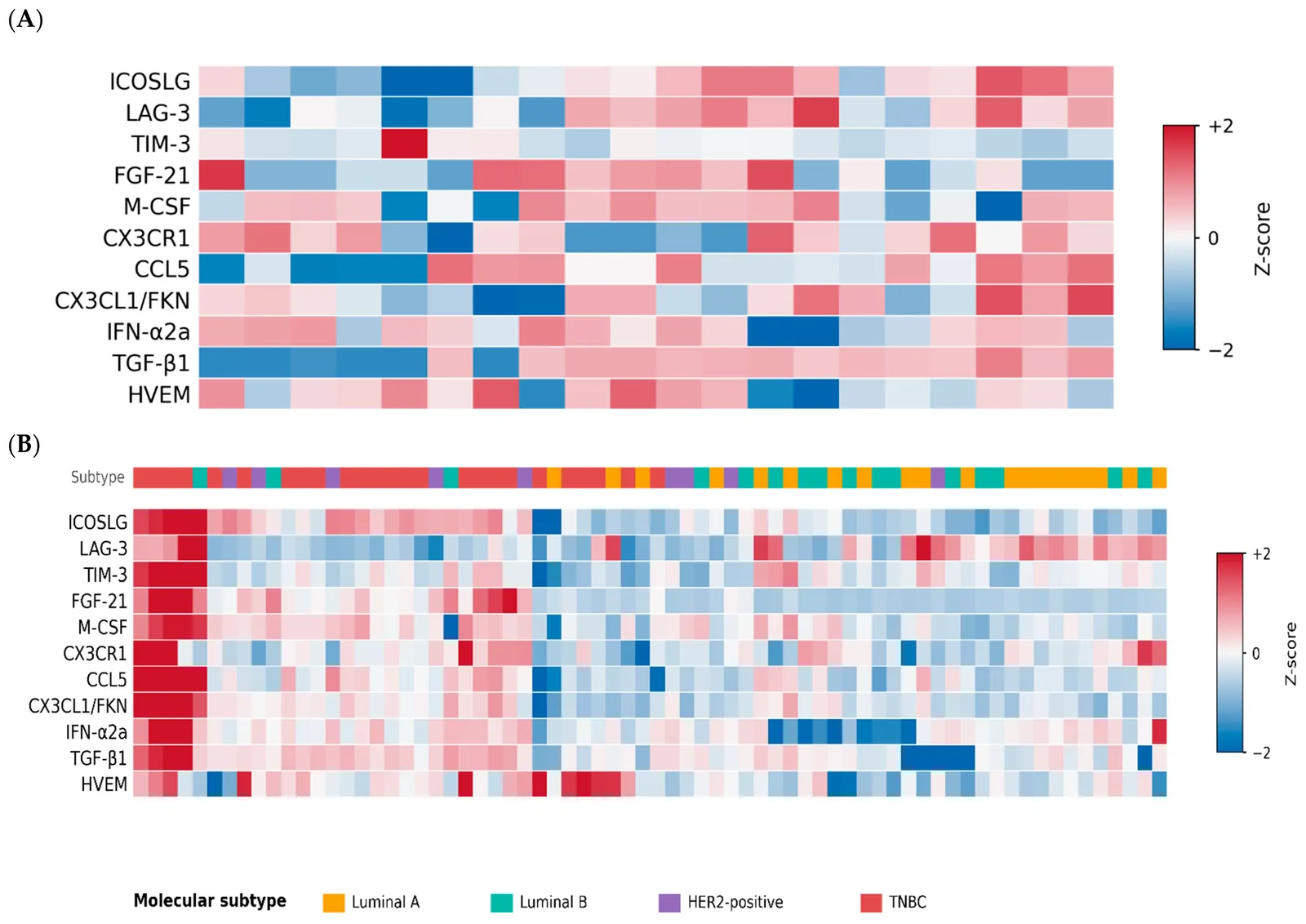
Comparative heatmaps of circulating immune biomarker profiles in healthy control women and early BC patients. (**A**) Healthy control women (*n* = 20). (**B**) Patients with early BC (*n* = 70).

The profiles of circulating immune biomarkers from healthy controls (*n* = 20) were compared with those of early BC patients (*n* = 70) using unsupervised hierarchical clustering (Figure 7). In the healthy control cohort, the immune profile exhibited notable homogeneity, with most biomarkers demonstrating low inter-individual variability in expression and clustering around the population mean across both low and high levels. Conversely, patients with BC exhibited significantly increased immune heterogeneity, characterized by substantial differences among patients in levels of soluble immune checkpoints, chemokines, growth factors, and cytokines. A higher expression pattern was observed in certain subsets of BC patients, with elevated levels of ICOSLG, TIM-3, M-CSF, CX3CR1, CCL5, CX3CL1/Fractalkine, and TNFRSF-14 (HVEM). In contrast, healthy controls showed more balanced expression patterns. Moreover, subtype classification revealed distinct immune phenotypes within the Luminal A, Luminal B, HER2-positive, and TNBC groups.

### 5.13. Principal Component Analysis of Circulating Immune Profiles

**Figure 8:**
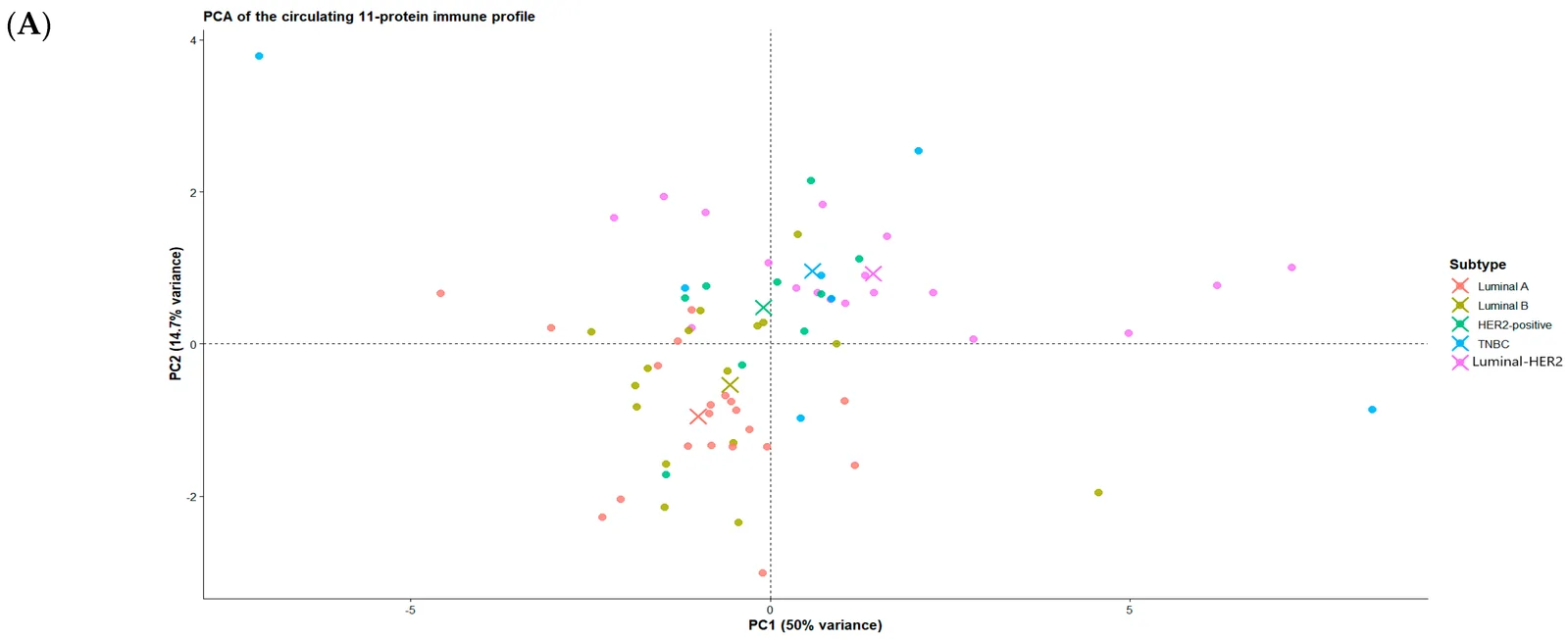

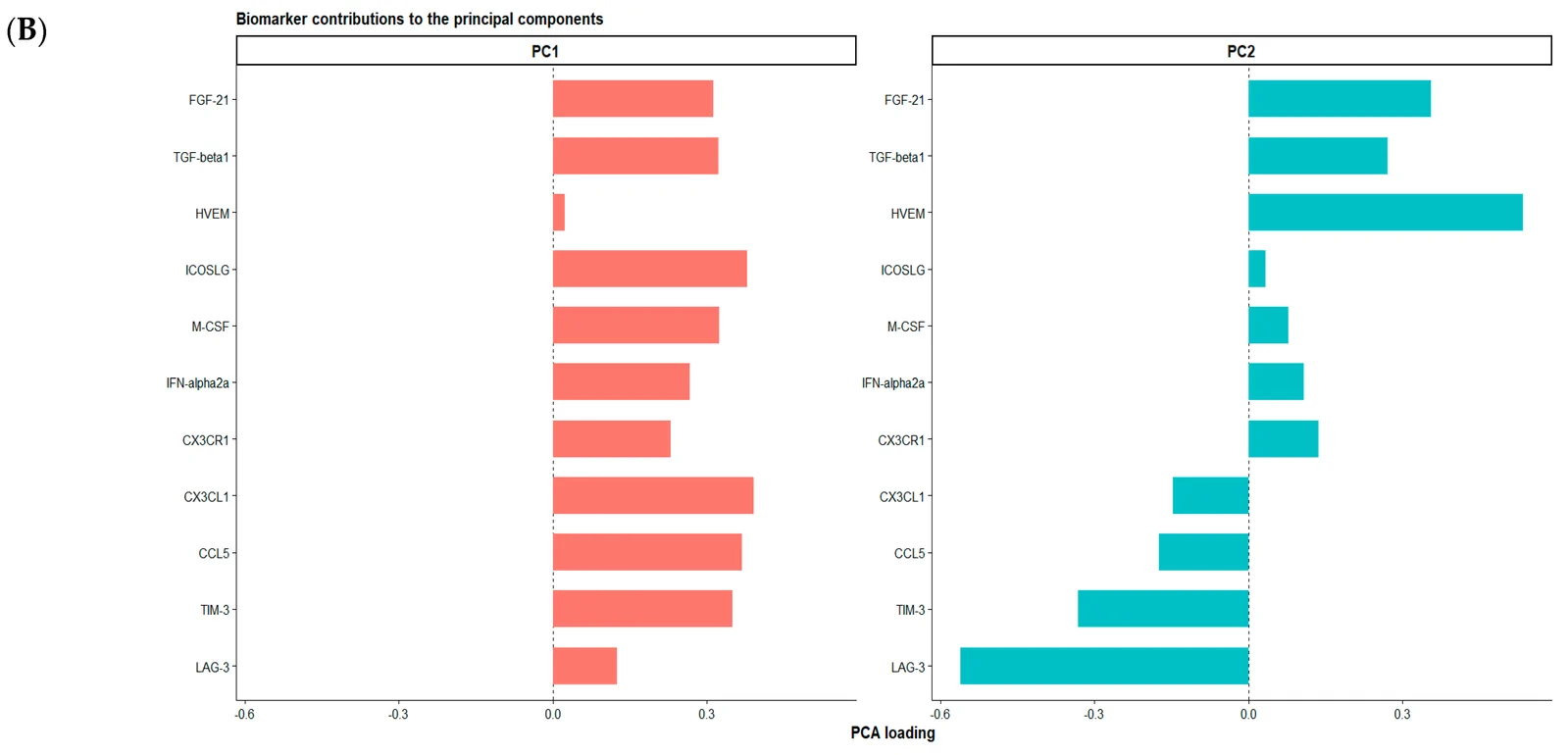
Multivariable circulating immune profile analysis and its relationship with conventional BC subtypes. (**A**) PCA of the standardized 11-protein circulating immune profile in 70 patients, colored according to conventional subtype. PC1 and PC2 accounted for 50.0% and 14.7% of total variance. (**B**) Biomarker loadings for PC1 and PC2.

Principal-component analysis (PCA) of the 11-protein panel identified a dominant multivariable pattern. PC1 accounted for 49.99% of the total variance, and PC2 explained 14.73%. The first two components together captured 64.71% of the overall variation in the circulating immune profile. The first three components accounted for 74.09% and the first five for 86.91% of total variance (Table 7, Figure 8A).

PC1 showed positive contributions from the whole biomarker panel, with the largest loadings for CX3CL1 (0.391), ICOSLG (0.379), CCL5 (0.369), TIM-3 (0.350), M-CSF (0.324), TGF-β1 (0.322), and FGF-21 (0.312). PC2 displayed a distinct loading pattern dominated by LAG-3 (−0.562) and HVEM (0.535), followed by FGF-21 (0.356), TIM-3 (−0.332), and TGF-β1 (0.270) (Figure 8B; Supplementary Table S2).

### 5.14. Relationships of the Principal Components with Clinicopathological Variables

PC1 was significantly associated with HER2 status, which remained significant after FDR correction (*p* = 0.00158; FDR = 0.01264). HER2-positive tumors had higher median PC1 scores than HER2-negative tumors (0.566 vs. −0.821), with a moderate effect size (r = 0.378; 95% CI, 0.17–0.59). PC1 also differed among conventional molecular subtypes (Kruskal–Wallis *p* = 0.004256; FDR = 0.01702; eta-squared[H] = 0.173). In post hoc Dunn testing, PC1 scores were significantly higher in the Luminal-HER2 group than in both Luminal A (adjusted *p* = 0.00661) and Luminal B (adjusted *p* = 0.01349).

PC2 showed stronger associations with receptor-defined tumor characteristics. HER2-positive tumors had a higher median PC2 score than HER2-negative tumors (0.732 vs. −0.748) (*p* = 2.209 × 10^−6^; FDR = 8.837 × 10^−6^; r = 0.566; 95% CI, 0.38–0.72). PC2 was also associated with ER status (*p* = 0.00642; FDR = 0.01712; r = 0.327; 95% CI, 0.09–0.52) and differed among molecular subtypes (*p* = 1.305 × 10^−6^; FDR = 8.837 × 10^−6^; eta-squared[H] = 0.443; 95% CI, 0.29–0.65). Although menopausal status showed a nominal PC2 association (*p* = 0.02788), this association did not remain significant after FDR correction (FDR = 0.05577).

For PC2, FDR-adjusted Dunn post hoc analysis identified significant differences between HER2-positive and Luminal A (adjusted *p* = 0.00644), HER2-positive and Luminal B (0.04466), Luminal A and TNBC (0.00486), Luminal B and TNBC (0.04375), Luminal A and Luminal-HER2 (5.29 × 10^−6^), and Luminal B and Luminal-HER2 (7.07 × 10^−4^) (Table 8).

### 5.15. Precision-Oncology and Public Multi-Omics Contextualization

At the genes/pathways level, the 11 proteins were categorized into four biological axes: T-cell co-regulation, chemokine trafficking, myeloid/metabolic growth, and cytokine regulation. According to Reactome mapping, the CX3CL1-CX3CR1 pair is involved in the process of chemokine-receptor binding and subsequent downstream G-alpha(i) signaling, whereas TNFRSF14 (HVEM) participates in BTLA-driven regulation of T-cell activation [18]. Importantly, these pathway annotations are presented as biological context and are not interpreted as evidence that these pathways were activated in the present cohort.

A targeted secondary in silico extension was then performed, using peer-reviewed analyses of publicly available BC resources, while avoiding the assumption that circulating protein concentrations are equivalent to tumor-tissue expression. TCGA multi-platform profiling revealed substantial heterogeneity across intrinsic BC groups, and METABRIC identified additional genomic/transcriptomic groups and immune-response networks across ~2000 tumors [19]. A separate TCGA/GEO analysis incorporated TNFRSF14 into an immune-related prognostic signature and reported its downregulation in BC tissues, together with associations between the prognostic risk groups and immune-cell infiltration [20]. Recent TCGA/UALCAN-based work also reported increased LAG3 transcript expression in TNBC compared to luminal or HER2-positive groups [21]. These tissue-level observations are not supposed to be equivalent to the soluble plasma concentrations measured in our study. Thus, differences between tissue-transcript and circulating-protein patterns underscore the importance of distinguishing biological compartment and biomarker modality.

**Figure 9:**
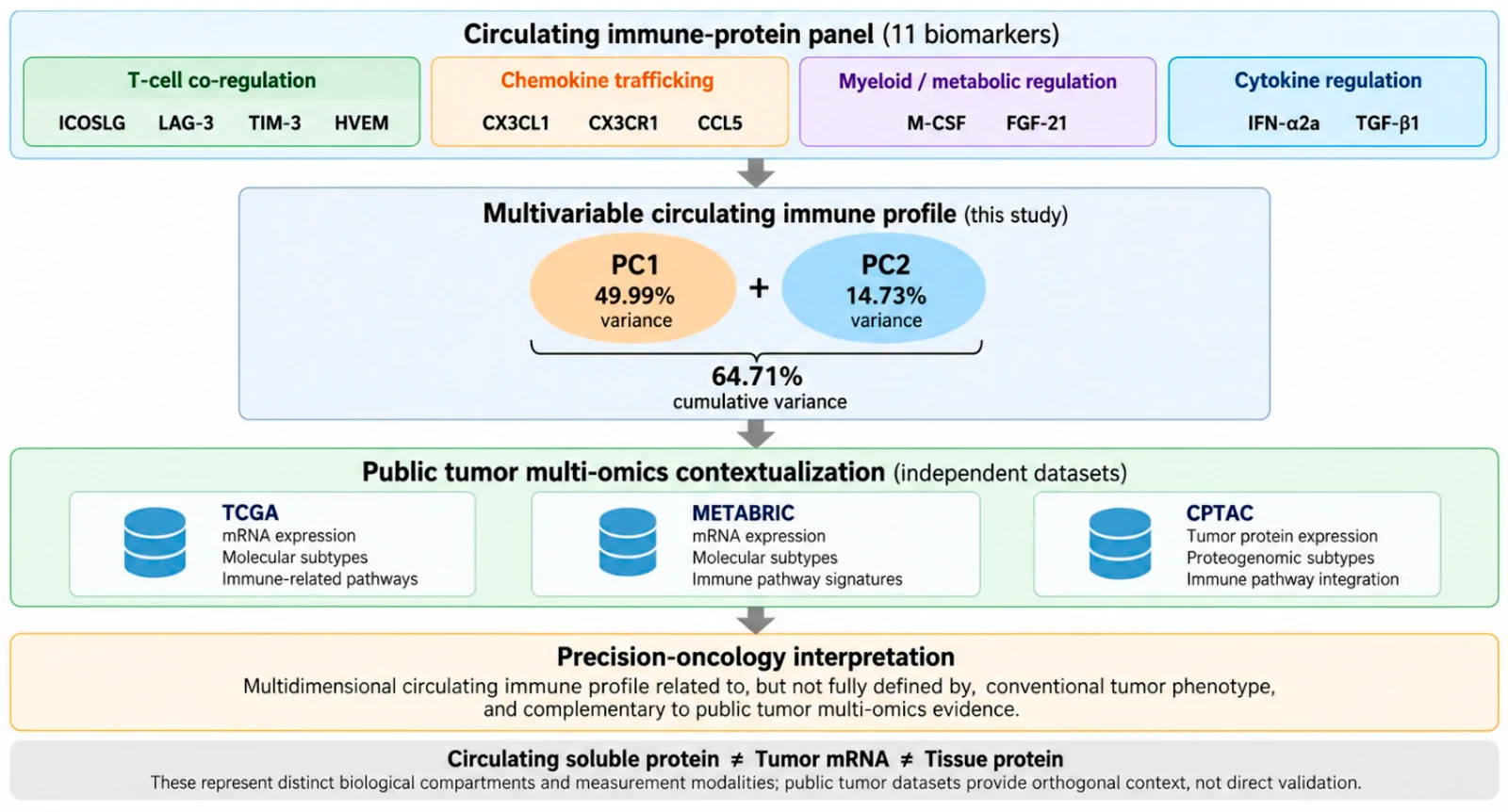
Precision-oncology framework integrating the 11-plasma protein panel with clinicopathological variables and public BC multi-omics resources.

A quantitative proteome study of BC demonstrated that immune-cell infiltration further distinguished basal-like and luminal B tumors, including an interferon-alpha-response-enriched luminal subset [22]. CPTAC proteogenomic analysis of 122 primary BC tumors naive to treatment integrated DNA/RNA with proteomic, phosphoproteomic, and acetylproteomic data and identified tumor subsets with distinct immune-therapy-relevant biology [23]. Taken together, these external datasets suggest that immune phenotypes may extend beyond conventional receptor-defined subgroups. However, they do not establish a direct correspondence between intratumoral mRNA or protein expression and the circulating plasma proteins measured in the present study (Figure 9).

## 6. Discussion

In the present study, we characterized plasma levels of circulating immune checkpoints, growth factors, chemokines, and cytokines in Egyptian women with early-stage (BC). The results showed systemic immune dysregulation, with elevated levels of ICOSLG, TIM-3, FGF-21, M-CSF, CCL5, CX3CL1/fractalkine, TGF-β1, and TNFRSF-14 (HVEM), along with a decreased level of LAG-3. To our knowledge, this is among the first studies to provide profiling of these soluble immune and inflammatory mediators in Egyptian women with early BC. More importantly, CX3CL1/fractalkine and TNFRSF-14 (HVEM) were identified as independent biomarkers associated with IHC-defined clinicopathological subgroups. Collectively, these findings suggest that early BC is associated with complex systemic immune and inflammatory changes.

The current study revealed a significant increase in plasma levels of ICOSLG in early BC patients compared with healthy controls. ICOSLG is a key co-stimulatory molecule that plays a critical role in T-cell activation and adaptive immune responses through binding to ICOS [24]. However, persistent activation of the ICOS/ICOSLG pathway may lead to an increase in regulatory T cell populations and immune tolerance [25]. Therefore, elevated circulating ICOSLG levels in our cohort may represent an antitumor immune response and immunoregulatory mechanisms. Consistent with our findings, activation or overexpression of the ICOS/ICOSLG pathway has been reported to be associated with BC progression and poorer outcomes [26,27]. Together with the simultaneous elevation of TIM-3, these findings suggest that immune activation and compensatory immune inhibition may be an early event in BC [4].

On the contrary, circulating LAG-3 levels were significantly reduced in BC patients compared with healthy controls. LAG-3 is another inhibitory immune checkpoint, similar to TIM-3, that plays a crucial role in maintaining immune system homeostasis and regulating T-cell immune response [28,29] The persistent expression of LAG-3 within the tumor microenvironment (TME) contributes to T-cell dysfunction and immune evasion [30]. In a large study by Liu et al., an increased LAG-3 tissue expression was reported in BC, particularly in the basal, HER2-positive, and Luminal A subtypes [31]. This study evaluated tissue expression, whereas our study focused on soluble LAG-3 circulating levels. The decreased plasma levels of LAG-3 observed in the current study reflect changes in LAG-3 expression within the TME, as suggested by previous studies [32,33]. This observation highlights the complexity of immune checkpoint regulation in BC. Our results demonstrated that both FGF-21 and M-CSF were significantly higher in BC patients compared with healthy controls. FGF-21 is an important metabolic regulator that links metabolic adaptation and cancer progression. Many studies reported that elevated levels of FGF-21 in BC patients may reflect the metabolic and oxidative stress associated with tumor growth [34] and suggested that it may have potential as a diagnostic biomarker [7,35,36]. These findings support the possible involvement of FGF-21 in the metabolic changes during BC development.

Similarly, plasma M-CSF levels were significantly increased in BC patients. M-CSF promotes differentiation of monocytes and recruitment of macrophages, contributing to the accumulation of tumor-associated macrophages (TAMs). Elevated M-CSF levels may therefore create an immunosuppressive microenvironment by promoting polarization of macrophages toward an M2 phenotype that favors tumor growth and angiogenesis [17]. Our findings may imply active macrophage-mediated immune remodeling in early-stage BC. Chemokines are important regulators of immune-cell trafficking and can mediate tumor progression by promoting immune cell migration, angiogenesis, and metastasis [16]. Similarly, the CX3CL1/CX3CR1 pathway has been linked to the survival of tumor cells and metastasis [37]. The present study reported concurrent elevation of CCL5, CX3CR1, and CX3CL1/fractalkine, suggesting active immune cell recruitment in early BC rather than immunological quiescence. Herein, the simultaneous high circulating levels of both ligand (CX3CL1) and receptor (CX3CR1) indicate activation of the CX3CL1/CX3CR1 pathway in early BC, which may contribute to modifying the systemic immune environment of BC.

In addition, TGF-β1 was significantly higher in the studied BC patients, while IFN-α2a showed no significant changes. TGF-β1 has well-known roles in modulating the immune response, epithelial-to-mesenchymal transition, and tumor progression [38]. Its increased levels in our study may therefore reflect an immune regulation that favors tumor persistence. Interestingly, circulating TNFRSF-14 (HVEM) levels were also markedly increased. TNFRSF-14 (HVEM) is a bidirectional immune regulator that can promote either stimulatory or inhibitory signals, depending on its interacting receptors [39]. The HVEM-BTLA pathway has been implicated in tumor immune tolerance [40], while targeting BTLA has been shown to enhance antitumor immune responses [41]. Thus, the elevated levels observed in this study may therefore reflect altered immune regulation, although this does not directly prove HVEM-mediated activity within the TME.

The simultaneous elevation of ICOSLG, TIM-3, TNFRSF-14 (HVEM), M-CSF, CCL5, CX3CL1, and TGF-β1 indicates that early BC is characterized by the coordinated activation of immune-regulatory pathways, rather than alterations in individual mediators alone. Collectively, these findings support a systemic immunosuppressive network involving T-cell exhaustion, macrophage activation, chemokine-mediated immune cell recruitment, and TGF-β-mediated immune modulation, consistent with previous literature [16,17,30,38]. Furthermore, IHC-defined clinicopathological subgrouping revealed biological heterogeneity among BC cases. Variations in ICOSLG, LAG-3, FGF-21, M-CSF, CX3CL1/fractalkine, TGF-β1, and TNFRSF-14 (HVEM). The data imply that the presence of different immune response patterns were observed between subgroups, suggesting distinct patterns of immune regulation. Specifically, Luminal-HER2 BC appeared to differ markedly from Luminal A in several biomarkers.

Furthermore, our findings suggest that CX3CL1/fractalkine and TNFRSF-14 (HVEM) may be associated with specific BC subtypes, particularly in distinguishing Luminal-HER2 from Luminal A. Although several biomarkers were significant in univariate analyses, only these two remained independently associated with the subtypes after adjusting for other variables. This suggests that CX3CL1/fractalkine and TNFRSF-14 (HVEM) may contribute to the immunological differences underlying BC heterogeneity [39,41]. These findings highlight their potential as non-invasive biomarkers for BC subtype stratification, although larger studies are needed for validation.

Notably, this study contributes to the limited data on immune profiles in Egyptian women diagnosed with BC, as most previous research has focused on European, North American, and East Asian populations [42,43]. Our findings suggest that systemic immune remodeling may occur early in BC and vary across IHC-defined clinicopathological subgroups. The identification of TNFRSF-14 (HVEM) and CX3CL1/fractalkine as independent circulating biomarkers highlights their potential as non-invasive biomarkers for BC subtype stratification and future precision immuno-oncology.

Multivariable analysis showed that the 11-protein panel reflects coordinated immune patterns beyond individual biomarkers. Two immune dimensions explained 64.7% of the total variance and were associated with HER2 status, ER status, and molecular subtype, although heterogeneity remained within each subtype. These findings suggest that BC immune variation is continuous rather than separated into distinct groups. Public multi-omics data added other context, but circulating proteins and tumor measurements represent different biological compartments.

Several limitations remain. First, it includes a relatively small sample size, particularly for the TNBC and HER2-positive subgroups, which may limit the statistical power of subgroup analyses and the generalizability of the findings. Second, only circulating soluble immune checkpoint molecules were evaluated, whereas membrane-bound expression in tumor tissues was not assessed. These limitations support prospective, paired tissue-blood validation in larger independent cohorts.

## 7. Conclusions

This exploratory study shows that early BC in Egyptian women is associated with systemic immune changes, involving immune checkpoints, chemokines, growth factors, and cytokines. Compared with healthy controls, patients showed higher levels of ICOSLG, TIM-3, FGF-21, M-CSF, CX3CR1, CCL5, CX3CL1/fractalkine, TGF-β1, and HVEM, alongside lower LAG-3 levels. Importantly, these immune changes varied across IHC-defined clinicopathological subgroups, with FGF-21, CX3CL1/fractalkine, and TNFRSF-14 (HVEM) particularly distinguishing Luminal-HER2 BC from the Luminal A subtype. CX3CL1/fractalkine and TNFRSF-14 (HVEM) retained independent associations with subtype, highlighting their potential as invasive biomarkers for subgroup stratification. These findings support that systemic immune dysregulation can be detected at an early stage of BC and may provide information complementary to conventional clinicopathological classification.

## Figures and Tables

**Table 1:** Patient characteristics.

Parameter		*n* = 70	(%)
Age	Mean (SD)	50	(12)
Menopausal Status	Pre-menopausal	35	(50.0)
	Peri-menopausal	3	(4.3)
	Post-menopausal	32	(45.7)
Grade	II	44	(62.9)
	III	26	(37.1)
Tumor size	T1	12	(17.1)
	T2	41	(58.6)
	T3	17	(24.3)
Lymph node	Negative	15	(21.4)
	Positive	55	(78.6)
ER	Negative	17	(24.3)
	Positive	53	(75.7)
PR	Negative	20	(28.6)
	Positive	50	(71.4)
HER2	Negative	41	(58.6)
	Positive	24	(34.3)
	Amplified	5	(7.1)
Ki-67	<14	29	(41.4)
	≥14	41	(58.6)
lymph vascular invasion Status	Absent	31	(44.3)
	Present	39	(55.7)
Family History of BC	Negative	36	(51.4)
	Positive	34	(48.6)
Histological type	Invasive Ductal Carcinoma	67	(95.7)
	Invasive Lobular Carcinoma	3	(4.3)
BC subtype	TNBC	7	(10.0)
	Luminal-HER2	18	(25.7)
	HER2- positive	9	(12.9)
	Luminal A	20	(28.6)
	Luminal B	16	(22.9)

**Table 2:** Comparison of plasma levels of soluble immune checkpoint molecules, growth factors, chemokines, and cytokines in patients with early-stage BC vs. healthy controls.

	Group				
	Early BC Cases (*n* = 70)		Healthy Controls (*n* = 20)		
	Median	(Range)	Median	(Range)	*p*-Value ^M^
Checkpoints					
ICOSLG	697.48	(406.98–1526.56)	510.25	(382.19–597.36)	<0.001 *
LAG-3	3.24	(1.48–19.31)	6.37	(3.84–9.08)	<0.001 *
TIM-3	350.40	(133.54–4796.19)	217.68	(157.46–4414.33)	<0.001 *
Growth Factors					
FGF-21	68.36	(59.20–2006.84)	57.49	(50.85–70.96)	<0.001 *
M-CSF	1635.51	(46.73–4613.77)	957.87	(436.05–1104.03)	<0.001 *
Chemokines					
CX3CR1	435.15	(190.10–2028.98)	277.44	(111.45–403.85)	<0.001 *
CCL5	484.26	(235.09–2558.21)	284.70	(145.38–480.46)	<0.001 *
CX3CL1-FKN	3.47	(0.97–20.14)	2.96	(1.95–3.70)	<0.001 *
Cytokines					
IFN-α2a	109.92	(54.24–484.70)	109.45	(60.24–126.71)	0.570
TGF-β1	451.78	(53.36–3229.25)	404.60	(53.45–717.87)	0.031 *
Dual					
TNFRSF-14 (HVEM)	266.12	(65.38–863.78)	219.73	(101.87–303.79)	0.002 *

* Statistically significant at *p*-value < 0.05 level. ^M^ Mann–Whitney U test.

**Table 3:** Circulating levels of the immune biomarkers across BC IHC-defined clinicopathological subgroups.

Biomarker	BC Subtype										
	TNBC		Luminal-HER2		HER2-Positive		Luminal A		Luminal B		
	Median	(Range)	Median	(Range)	Median	(Range)	Median	(Range)	Median	(Range)	*p*-Value ^K^
Checkpoints											
ICOSLG	823.58	(406.98–1526.56)	840.45	(582.72–1244.96)	769.11	(591.37–903.16)	645.37	(417.47–796.55)	650.71	(530.08–1244.37)	0.007 *
LAG-3	2.73	(1.73–16.94)	3.05	(1.60–6.50)	2.63	(1.48–7.64)	5.93	(2.20–11.49)	3.93	(2.21–19.31)	0.012 *
TIM-3	382.52	(133.54–4796.19)	350.40	(208.06–1469.98)	285.70	(235.62–451.06)	353.97	(178.94–643.49)	343.60	(228.17–1043.75)	0.563
Growth Factors											
FGF-21	98.58	(63.95–2006.84)	106.50	(62.55–1186.75)	93.39	(61.66–147.62)	63.61	(59.21-72.63)	62.99	(59.20–199.87)	<0.001 *
M-CSF	1948.94	(1229.03–4596.01)	1817.83	(1309.73–4613.77)	1795.47	(1248.90–2007.08)	1355.84	(597.73–2527.67)	1393.11	(46.73–4008.99)	0.003 *
Chemokines											
CX3CR1	436.82	(299.48–2028.98)	498.40	(276.23–1465.07)	388.58	(293.31–677.86)	410.29	(190.10–775.98)	424.42	(272.31–909.11)	0.122
CCL5	484.90	(235.09–2558.21)	561.20	(407.56–1525.72)	469.93	(395.75–755.55)	466.57	(277.05–660.69)	445.31	(325.32–1404.94)	0.073
CX3CL1-FKN	3.62	(0.97–20.14)	4.00	(2.73–12.01)	3.41	(2.57–3.99)	3.19	(2.22–4.86)	3.38	(2.71–6.33)	0.016 *
Cytokines											
IFN-α2a	115.24	(72.12–484.70)	106.24	(90.41–218.52)	118.85	(100.70–133.49)	111.33	(54.24–191.42)	104.29	(57.16–129.07)	0.105
TGF-β1	511.97	(186.05–3229.25)	616.66	(298.95–2308.93)	478.46	(53.80–589.27)	347.21	(54.18–596.26)	441.47	(53.36–752.07)	<0.001 *
Dual											
TNFRSF-14 (HVEM)	268.34	(65.38–863.78)	362.91	(227.34–741.33)	276.14	(174.23–389.75)	244.84	(120.30–583.39)	233.33	(124.38–350.27)	0.001 *

^K^ Kruskal–Wallis test. * Statistically significant at *p*-value < 0.05 level.

**Table 4:** Pairwise post hoc comparisons of significantly different circulating biomarkers across BC IHC-defined clinicopathological subgroups employing the Bonferroni correction.

Biomarker	Comparison	Unadjusted *p*-Value	Adjusted *p*-Value
ICOSLG	Luminal A vs. Luminal-HER2	0.009	0.093
	Luminal B vs. Luminal-HER2	0.039	0.393
LAG-3	HER2-positive vs. Luminal A	0.014	0.143
	TNBC vs. Luminal B	0.033	0.332
	TNBC vs. Luminal A	0.037	0.372
	Luminal-HER2 vs. Luminal A	0.009	0.093
FGF-21	Luminal B vs. TNBC	0.016	0.161
	Luminal B vs. Luminal-HER2	0.006	0.060
	Luminal A vs. TNBC	0.021	0.208
	Luminal A vs. Luminal-HER2	<0.001	0.001 *
M-CSF	Luminal A vs. HER2-positive	0.033	0.329
	Luminal A vs. Luminal-HER2	0.009	0.093
	Luminal A vs. TNBC	0.021	0.208
	Luminal B vs. HER2-positive	0.025	0.254
	Luminal B vs. Luminal-HER2	0.006	0.060
	Luminal B vs. TNBC	0.016	0.161
CX3CL1/Fractalkine	Luminal A vs. Luminal-HER2	0.001	0.012 *
	Luminal B vs. Luminal-HER2	0.006	0.060
	HER2-positive vs. Luminal-HER2	0.006	0.065
TGF-β1	Luminal A vs. Luminal B	0.044	0.442
	Luminal A vs. HER2-positive	0.033	0.329
	Luminal A vs. TNBC	0.021	0.208
	Luminal A vs. Luminal-HER2	0.009	0.093
	Luminal B vs. TNBC	0.016	0.161
	Luminal B vs. Luminal-HER2	0.006	0.060
	HER2-positive vs. Luminal-HER2	0.006	0.065
TNFRSF-14 (HVEM)	Luminal B vs. Luminal-HER2	0.006	0.060
	Luminal A vs. Luminal-HER2	<0.001	<0.001 *

* Statistically significant at *p*-value < 0.05 level.

**Table 5:** Multivariate logistic regression analysis of independent biomarkers discriminating Luminal-HER2 BC from Luminal A BC subtype.

Biomarker	B	S.E.	*p*-Value	OR	95% C.I. (OR)	
					Lower Limit	Upper Limit
CX3CL1-FKN	−3.367	1.233	0.006 *	0.034	0.003	0.387
Dual TNFRSF-14 (HVEM)	−0.019	0.007	0.005 *	0.981	0.969	0.994
Constant	18.063	5.942	0.002	69,952,385.771		

B: Regression coefficient, S.E.: Standard error, OR: Odds Ratio, CI: Confidence interval. * Statistically significant at *p*-value < 0.05.

**Table 6:** Correlations between clinicopathological features of the studied participants and the analyzed biomarkers.

Biomarker	Age (r, *p*-Value)	Menopause		Grade	Tumor Size	ER		PR		Family History	HER2
		Post Menopausal (*n* = 32) Median (Range)	Pre/Peri Menopausal (*n* = 38) Median (Range)			ER-Positive (*n* = 53)	ER-Negative (*n* = 17)	PR-Positive (*n* = 50)	PR-Negative (*n* = 20)		
		(*p*-Value ^M^)				(*p*-Value ^M^)		(*p*-Value ^M^)			
ICOSLG	−0.011, 0.929	NS		NS	NS	NS		NS		NS	0.003 *
LAG-3	−0.021, 0.864	NS		NS	NS	0.010 *		NS		NS	0.016 *
TIM-3	0.040, 0.740	NS		NS	NS	NS		NS		NS	NS
FGF-21	−0.170, 0.159	NS		NS	NS	0.018 *		NS		NS	<0.001 *
M-CSF	−0.029, 0.811	NS		NS	NS	0.016 *		NS		NS	0.001 *
CX3CR1	−0.103, 0.395	NS		NS	NS	NS		NS		NS	NS
CCL5	0.157, 0.194	NS		NS	NS	NS		NS		NS	0.029 *
CX3CL1/Fractalkine	−0.075, 0.538	NS		NS	NS	NS		NS		NS	0.024 *
IFN-α2a	0.018, 0.881	NS		NS	NS	0.037 *		0.015 *		NS	NS
TGF-β1	0.064, 0.597	NS		NS	NS	NS		NS		NS	0.007 *
TNFRSF-14 (HVEM)	−0.184, 0.128	0.020 *		NS	NS	NS		NS		NS	<0.001 *

r: Spearman’s rho Correlation Coefficient, ^M^ Mann–Whitney U test. * Statistically significant at *p*-value < 0.05. NS means non-significant.

**Table 7:** Variance explained by principal components derived from the circulating 11-protein immune profile.

Component	Variance Explained (%)	Cumulative Variance (%)
PC1	49.99	49.99
PC2	14.73	64.71
PC3	9.38	74.09
PC4	6.65	80.75
PC5	6.17	86.91
PC6	3.97	90.88
PC7	3.32	94.20
PC8	2.40	96.60
PC9	1.56	98.15
PC10	1.05	99.20
PC11	0.80	100.00

**Table 8:** Associations of PC1 and PC2 with clinicopathological characteristics.

Variable	PC1 P	PC1 FDR	PC2 P	PC2 FDR
ER	0.1624	0.3024	0.00642	0.01712
PR	0.3013	0.4018	0.1142	0.1762
HER2	0.00158	0.01264	2.209 × 10^−6^	8.837 × 10^−6^
Grade	0.1155	0.3024	0.5077	0.5802
Lymph-node status	0.9202	0.9202	0.8636	0.8636
Menopausal status	0.1890	0.3024	0.02788	0.05577
Molecular subtype	0.004256	0.01702	1.305 × 10^−6^	8.837 × 10^−6^

## Data Availability

All data generated are present in the current manuscript.

## Supplementary Materials

The following supporting information can be downloaded at: https://www.mdpi.com/article/10.3390/cells15201849/s1, Figure S1: Biomarker distributions across IHC-defined groups and healthy controls; Table S1: Available background information for healthy controls; Table S2: PC1 and PC2 biomarker loading matrix.

## Abbreviations

Abbreviations	Full Term
AUC	Area Under the Curve
BC	Breast Cancer
CCL5	C-C Motif Chemokine Ligand 5
CI	Confidence Interval
CX3CL1/Fractalkine	C-X3-C Motif Chemokine Ligand 1 (Fractalkine)
CX3CR1	C-X3-C Motif Chemokine Receptor 1
ER	Estrogen Receptor
FGF-21	Fibroblast Growth Factor 21
HER2	Human Epidermal Growth Factor Receptor 2
TNFRSF-14 (HVEM)	Herpesvirus Entry Mediator
ICOSLG	Inducible T-cell Co-Stimulator Ligand
IFN-α2a	Interferon Alpha-2a
LAG-3	Lymphocyte Activation Gene-3
M-CSF	Macrophage Colony-Stimulating Factor
OR	Odds Ratio
PR	Progesterone Receptor
ROC	Receiver Operating Characteristic
SE	Standard Error
TAM	Tumor-Associated Macrophage
TGF-B1	Transforming Growth Factor Beta 1
TIM-3	T-cell Immunoglobulin and Mucin-domain Containing-3
TME	Tumor Microenvironment
TNBC	Triple-Negative Breast Cancer
TNFRSF14	Tumor Necrosis Factor Receptor Superfamily Member 14
Z-score	Standardized Score
