# Supplementary material for: Circulating Immune-Related Biomarkers in Egyptian Women with Early Breast Cancer: An Exploratory Case–Control Study Across IHC-Defined Clinicopathological Subgroups
Source: Cells. 2026 Oct 12;15(20):1849. doi: 10.3390/cells15201849 (PMC13703832; doi:10.3390/cells15201849)
Supplement: Supplementary file 1 [file cells-15-01849-s001.zip › cells-4573435-supplementary.pdf]

Supplementary Material

Circulating Immune-Related Biomarkers in Egyptian Women with Early Breast Cancer: An Exploratory Case–Control Study Across Molecular Subtypes.

Supplementary Table S1. Available background information for healthy controls

Variable	Available information
Number	20
Sex	Female
Age	Age-matched to the BC cohort<br>Mean (46)
History of malignancy	Excluded
Autoimmune disease	Excluded
Chronic inflammatory disorders	Excluded
Active infection	Excluded

Supplementary Table S2. PC1 and PC2 biomarker loading matrix

Biomarker	PC1 loading	PC2 loading
ICOSLG	0.379	0.033
LAG-3	0.125	-0.562
TIM-3	0.350	-0.332
FGF-21	0.312	0.356
M-CSF	0.324	0.077
CX3CR1	0.229	0.136
CCL5	0.369	-0.174
CX3CL1	0.391	-0.147
IFN-alpha2a	0.267	0.108
TGF-beta1	0.322	0.270
HVEM	0.023	0.535

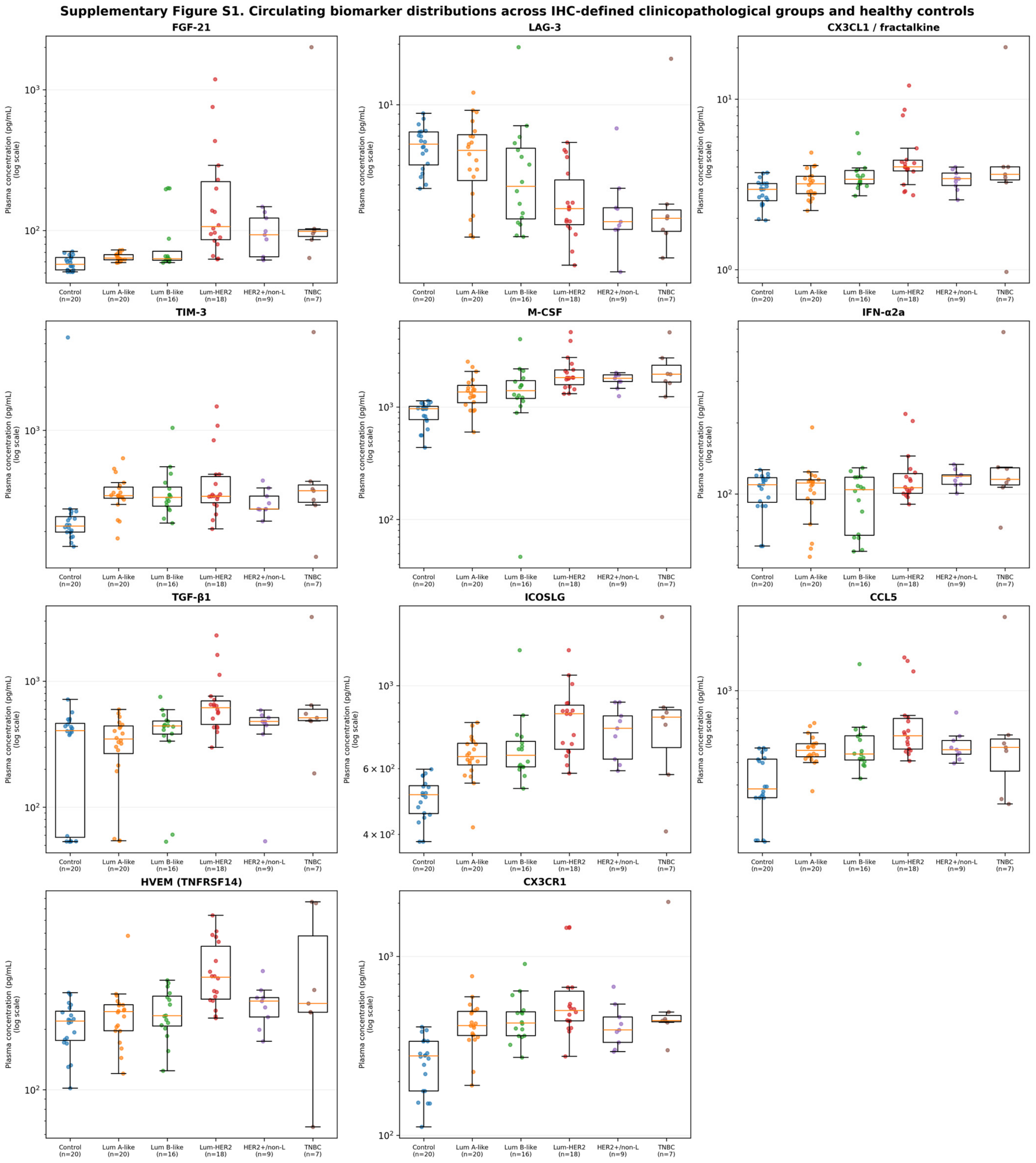

Supplementary Figure S1. Biomarker distributions across IHC-defined groups and healthy controls

Box plots with individual observations for all 11 measured circulating proteins in healthy controls (n = 20), Luminal A-like (n = 20), Luminal B-like (n = 16), Luminal-HER2-like (n = 18), HER2-positive (n = 9), and TNBC (n = 7). Y-axes are logarithmic because several analytes show strongly right-skewed distributions. The figure is descriptive and does not by itself imply equivalence between these IHC-defined clinicopathological groups and PAM50 intrinsic molecular subtypes.
